# Comparison of the C—H⋯O bonding in two crystalline phases of 1,4-di­thiane 1,1,4,4-tetra­oxide

**DOI:** 10.1107/S2056989019004407

**Published:** 2019-04-05

**Authors:** Richard L. Harlow, Allen G. Oliver, Jonathan M. Baker, William J. Marshall, Michael P. Sammes

**Affiliations:** a 838 Grooms Rd, Rexford, NY 12148, USA; b Dept. of Chemistry and Biochemistry, University of Notre Dame, Notre Dame, IN 46556-5670, USA; c DuPont Experimental Station E500.com, 200 Powder Mill Road, PO Box 8352, Wilmington, DE 19803, USA; d 2 Baydons Lane, Chippenham SN15 3JX, UK

**Keywords:** crystal structures, C—H⋯O bonding, 1,4-di­thiane-1,1,4,4-tetra­oxide

## Abstract

The crystal structures of two crystalline phases of 1,4-di­thiane 1,1,4,4-tetra­oxide, C_4_H_8_O_4_S_2_, have been determined in order to examine the nature of possible inter­molecular hydrogen bonds. Phase 1 is monoclinic, space group *C*2/*m*, with unit-cell dimensions of *a* = 9.073 (8), *b* = 7.077 (6), *c* = 5.597 (5) Å and β = 105.89 (1)°. The mol­ecule adopts 2/*m* symmetry and all of the mol­ecules are related by translation and thus have the same orientation. Phase 2 is also monoclinic but in space group *P*2_1_/*n* with unit-cell dimensions of *a* = 7.1305 (5), *b* = 5.7245 (4), *c* = 8.3760 (6) Å and β = 91.138 (2)°. In this phase, the mol­ecule sits on an inversion center and the mol­ecules within the unit cell adopt quite different orientations. In both phases, examination of the potential C—H⋯O hydrogen bonds around each of the independent oxygen atoms (one axial and the other equatorial) shows the general O⋯H patterns to be quite similar with each oxygen atom in contact with four neighboring H atoms, and each H atom contacting two neighboring O atoms. While none of the H⋯O contacts is particularly short (all are greater than 2.5 Å), each mol­ecule has 32 such contacts that form an extensive inter­molecular network. A ^1^H NMR spectrum of the compound dissolved in DMSO shows a singlet of 8H at δ 3.677 which indicates that the C—H bonds are only moderately polarized by the single adjacent –SO_2_– moiety: strongly polarized C—H bonds have δ values in the 5–6 range [Li & Sammes (1983 ▸). *J. Chem. Soc. Perkin Trans. 1*, pp. 1303–1309]. The phase 1 crystal studied was non-merohedrally twinned.

## Abstract 2

The structures of two crystalline phases of 1,4-di­thiane-1,1,4,4-tetra­oxide have been determined and found to have similar local C—H⋯O hydrogen-bonding arrangements in spite of differences in the mol­ecular packing.

## Chemical context   

Some years ago, multiple studies of C—H⋯*X* (*X* = N, O) *intra*mol­ecular hydrogen bonds were carried out on a series of 1,3-di­thiane 1,1,3,3-tetra­oxides which had various substituents at the 2 position located between the two SO_2_ groups. The remaining C—H bond in the 2 position is strongly polarized given the electron-withdrawing properties of the two adjacent sulfone groups. The substituents bonded at the 2 position contained nitro­gen or oxygen electron-pair donors which, with proper chain lengths, were able to form an intra­molecular hydrogen bond to the polar hydrogen atom.
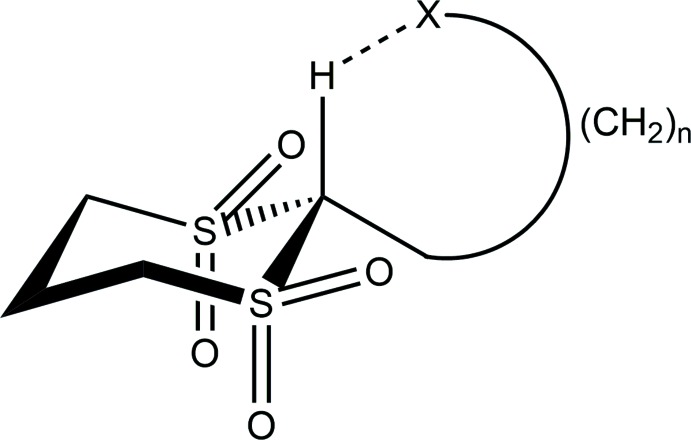


The chemistry and NMR/IR spectroscopic information of a wide variety of compounds were reported in a number of papers (see Li & Sammes, 1983 ▸, and references therein). Focusing on those compounds with significant shifts of the polar methine hydrogen in the ^1^H-NMR spectra, their crystal structure determinations clearly demonstrated the formation of *intra*mol­ecular hydrogen bonds (Harlow *et al.*, 1984 ▸). Never explored, however, was the nature of the C—H⋯O inter­actions likely to be found in the unsubstituted compound itself. As a matter of curiosity, we consequently decided to undertake the crystal structure determinations of the two possible (1,3- and 1,4-) di­thiane tetra­oxides and the unique 1,3,5-tri­thiane hexa­oxide. All three of the compounds have unusually high melting/decomposition temperatures and we wanted to explore and compare the nature of the *inter*mol­ecular C—H⋯O inter­actions in this group of uncomplicated compounds. As a start on this project, we report herein the completion of the structures of two crystalline phases of 1,4-di­thiane 1,1,4,4-tetra­oxide, a compound which has no dipole moment, has the same 1:2 O:H ratio as water, and decomposes above 627 K.
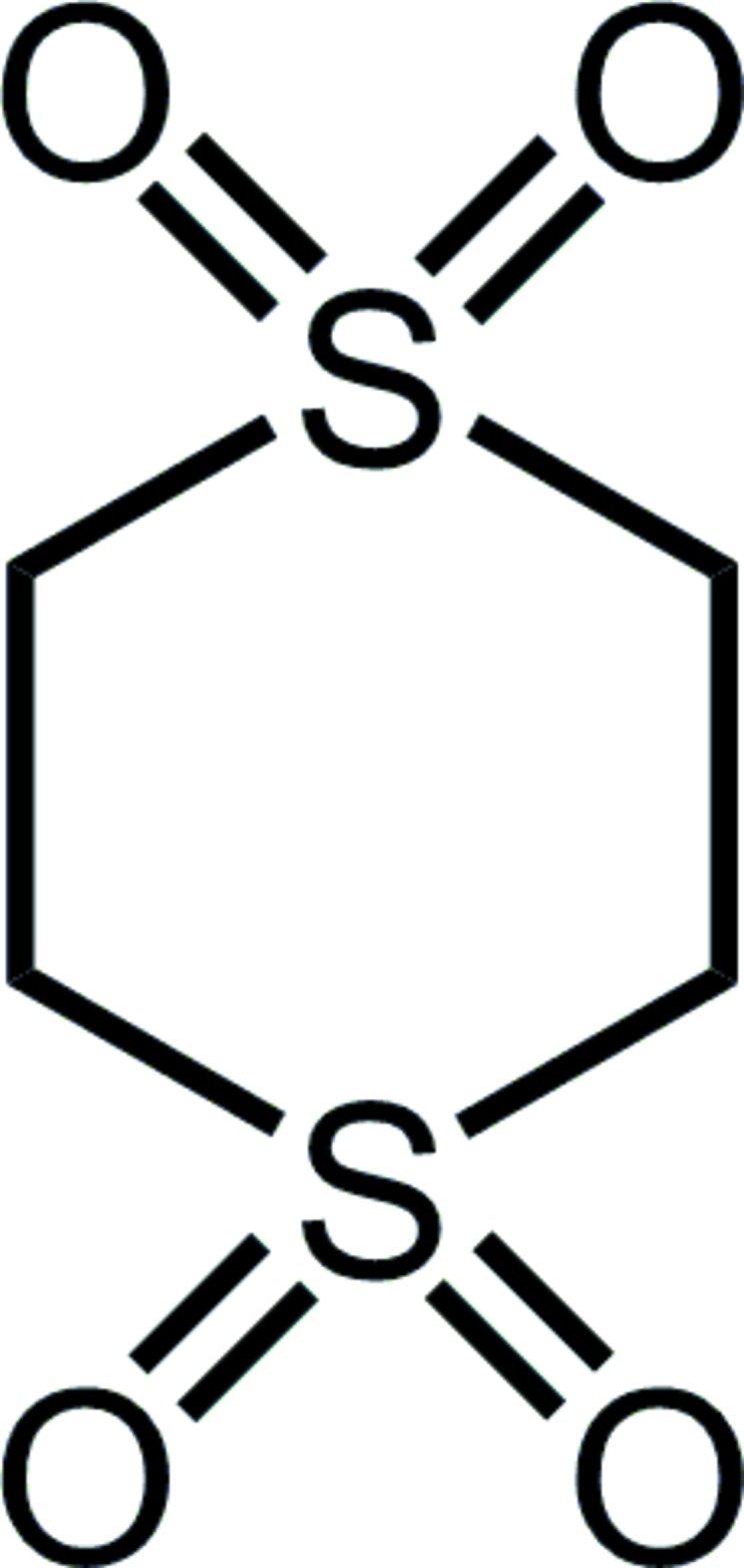


## Structural commentary   

**Figure 1:**
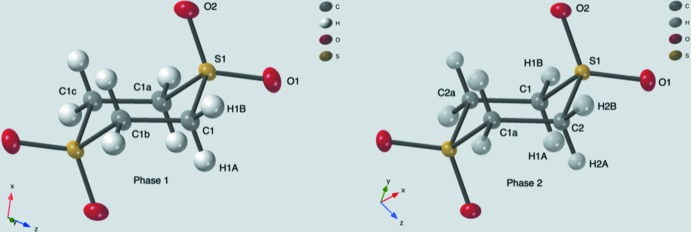
*ORTEP* drawings (50% probability) of the 1,4-di­thiane 1,1,4,4-tetra­oxide mol­ecule in crystalline phases 1 and 2. The mol­ecule in phase 1 has 2/*m* symmetry; in phase 2, it has a center of inversion. All of the unique atoms are labeled as are the symmetry-related carbon atoms to emphasize the different symmetries. Symmetry codes for phase 1: (a) *x*, 1 − *y*, *z*; (b) 1 − *x*, *y*, −*z*; (c) 1 − *x*, 1 − *y*, −*z*. Symmetry code for phase 2: (a) −*x*, −*y*, 1 − *z*.

1,4-Di­thiane 1,1,4,4-tetra­oxide contains two crystalline phases as determined from an X-ray diffraction pattern of the as-synthesized powder. When sublimed, crystals of both phases were also produced and it was only by chance that the two laboratories involved picked different phases. Fig. 1 ▸ compares the mol­ecular *ORTEP* drawings of the mol­ecules in the two phases. The mol­ecule in phase 1 adopts 2/*m* symmetry while in phase 2 the mol­ecule sits on a center of symmetry. The intra­molecular bond distances and angles for the two phases are comparable.

## Supra­molecular features   

**Figure 2:**
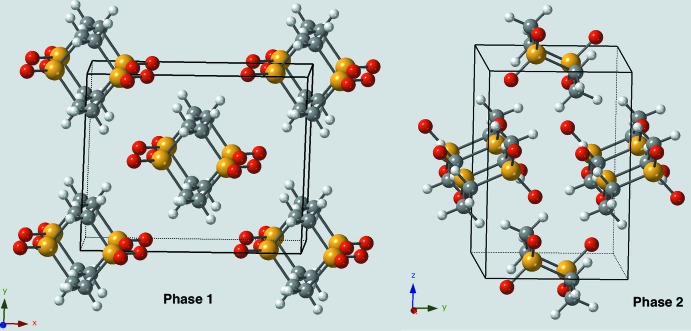
Packing diagrams for crystalline phase 1 as viewed nearly along the *c* axis and phase 2 as viewed nearly along the *a*-axis. In phase 1, all the mol­ecules are related by translation and thus have the same orientation. In phase 2, the mol­ecules have two different orientations.

**Figure 3:**
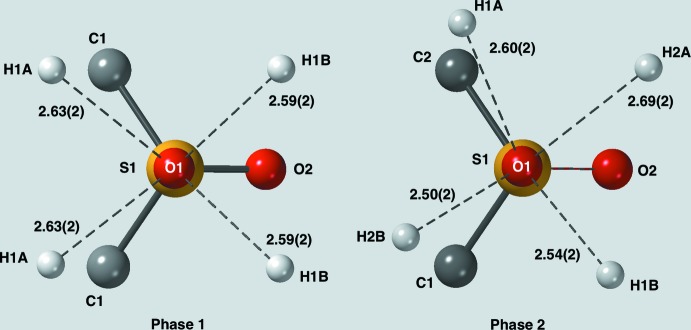
Environment of the equatorial oxygen atom, O1, in phases 1 and 2. Although the packing of the mol­ecules is quite different, the arrangement of the C—H⋯O contacts in both phases is seen to be very similar.

Packing diagrams (Fig. 2 ▸) reveal that the packing for the two forms is quite different. In phase 1, all of the mol­ecules are related by simple translational symmetries and thus all the mol­ecules have the same orientation. In phase 2, the mol­ecules have two different orientations in a somewhat herringbone fashion. Thus, one might expect any C—H⋯O contacts to be quite different for the two phases but, in fact, they are very similar. Figs. 3 ▸ and 4 ▸ compare the environments of O1 (equatorial oxygen atom) and O2 (axial oxygen atom). In all cases, each oxygen atom is in contact with four hydrogen atoms arranged in a distorted square. Probably for steric reasons, the distortion is less for the equatorial oxygen atom than for the axial oxygen atom.

Each oxygen atom in both phases ‘sees’ four hydrogen atoms while each hydrogen atom ‘sees’ two oxygen atoms. This bifurcation of the hydrogen contacts means that none of the H⋯O distances is particularly short. It should also be pointed out that each methyl­ene group has only one neighboring sulfone group, which would limit the polarization of the C—H bonds compared to our previous studies where the C—H bond of inter­est sat between two sulfone groups. Thus, very short C—H⋯O bonds were not expected. The exact details of the C—H⋯O contacts are given in Table 1 ▸. Thus, while there are no really short C—H⋯O contacts (none less than 2.50 Å), every donor and every acceptor plays a role in forming a extensive network of contacts in which each mol­ecule has a total of 32 inter­actions with its neighbors.

The shortest C—H⋯O contacts tend to be between the equatorial oxygen atoms, O1, and the equatorial hydrogen atoms labeled with the suffix *B*. These also come with C—H⋯O angles that are closest to being linear, 148 to 160°. Presumably the difference between axial and equatorial H⋯O contacts is mostly due to steric effects, the equatorial atoms being more accessible. The shorter contacts can undoubtedly be classified as true C—H⋯O hydrogen bonds using, as a guide, the seminal study of weak hydrogen bonds by Desiraju & Steiner (1999 ▸). The remaining bonds are probably better described as mostly electrostatic in nature. However, as Desiraju & Steiner point out, there are no hard limits for determining what may, and may not, be a true hydrogen bond.

## Database survey   

A Cambridge Crystallographic Database survey of 1,4-di­thiane reveals over 200 structures with that base motif (CSD v. 5.40 + 1 update; Groom *et al.*, 2016 ▸). A more modest survey, with one oxygen bonded to each sulfur yields 33 results, of which 1,4-di­thiane 1,4-dioxide has two polymorphs [DTHDOX and DTHDOX01 (Shearer, 1959 ▸; Takemura *et al.*, 2014 ▸) and DTHDSX (Montgomery, 1960 ▸)]. There is only one reported structure that incorporates 1,4-di­thiane 1,1,4,4-tetra­oxide into its structure, *viz. *5,6,7-triphenyl-2,3-di­hydro-6*H*-phospholo[3,4-*b*][1,4]dithiine 1,1,4,4,6-penta­oxide (GACCUK; Fadhel *et al.*, 2010 ▸). One of the five oxygen atoms is located on the phospho­rus, while the remaining four are on the sulfur atoms of the sulfone moiety.

## Synthesis and crystallization   

Following literature procedures (Schultz *et al.*, 1963 ▸), a 100 mL round-bottom flask was charged with 1,4-thiane (Sigma–Aldrich; 1.005 g, 8.4 mmol) in 25 mL glacial acetic acid. To this were added 10 mL 30% hydrogen peroxide solution (excess) in 25 mL of glacial acetic acid. The solution was heated to 323 K for 12 h under stirring over an oil bath. The white solid that formed was filtered and washed with water (3 × 25 mL) and diethyl ether (3 × 25 mL) (yield: 1.325 g, 86%). Crystals suitable for structural analysis were grown by sublimation of the solid. NMR data were recorded on a Bruker Avance 400 MHz with *d*_6_-DMSO as solvent, referenced to residue proteo-DMSO. TGA/DSC data showed decomposition occurring from 627 to 739 K.

## Refinement   

Crystal data, data collection and structure refinement details are summarized in Table 2 ▸. The refinement of phase 1 used two sets of reflections as the crystal was non-merohedrally twinned. Non-hydrogen atoms were refined with anisotropic atomic displacement parameters. All hydrogen atoms were refined freely.

## Supplementary Material

Crystal structure: contains datablock(s) 14-disulphone-phase1, 14-disulphone-phase2, global. DOI: 10.1107/S2056989019004407/jj2210sup1.cif

Structure factors: contains datablock(s) 14-disulphone-phase1. DOI: 10.1107/S2056989019004407/jj221014-disulphone-phase1sup2.hkl

Structure factors: contains datablock(s) 14-disulphone-phase2. DOI: 10.1107/S2056989019004407/jj221014-disulphone-phase2sup3.hkl

Supporting information file. DOI: 10.1107/S2056989019004407/jj221014-disulphone-phase1sup4.cml

Supporting information file. DOI: 10.1107/S2056989019004407/jj221014-disulphone-phase2sup5.cml

CCDC references: 1907090, 1907089

Additional supporting information:  crystallographic information; 3D view; checkCIF report

## Figures and Tables

**Figure 4:**
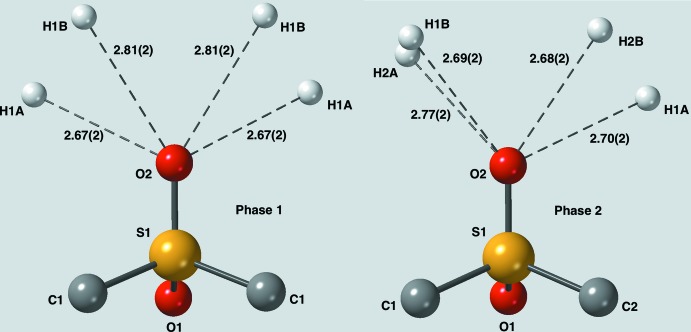
Environment of the axial oxygen atom, O2, in phases 1 and 2. In this case, the environments are still similar, but less so than for equatorial O1.

**Table 1:** Inter­molecular contacts (Å, °) as potential C⋯H⋯O h*y*drogen bonds for phases 1 and 2

Atoms	bondH⋯O	angleC—H⋯O	angleS—O⋯H
Phase 1			
C1—H1*A*⋯O1^i^	2.63 (2)	148 (2)	123 (2)
C1—H1*B*⋯O1^ii^	2.59 (2)	157 (2)	127 (2)
			
C1—H1*A*⋯O2^iii^	2.67 (2)	112 (2)	114 (2)
C1—H1*B*⋯O2^iv^	2.81 (2)	122 (2)	145 (2)
			
Phase 2			
C1—H1*A*⋯O1^i^	2.60 (2)	151 (1)	123 (1)
C1—H1*B*⋯O1^ii^	2.54 (2)	160 (1)	127 (1)
C2—H2*A*⋯O1^i^	2.69 (2)	149 (1)	122 (1)
C2—H2*B*⋯O1^iii^	2.50 (2)	155 (1)	129 (1)
			
C1—H1*A*⋯O2^iv^	2.70 (2)	110 (1)	111 (1)
C1—H1*B*⋯O2^v^	2.69 (2)	122 (1)	140 (1)
C2—H2*A*⋯O2^vi^	2.77 (2)	100 (1)	127 (1)
C2—H2*B*⋯O2^vii^	2.68 (2)	125 (1)	144 (1)

Symmetry codes for phase 1: (i) 1 − *x*, *y*, 1 − *z*; (ii) 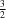 − *x*, −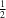 + *y*, 1 − *z*; (iii) −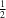 + *x*, −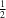 + *y*, *z*; (iv) 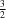 − *x*, −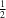 + *y*, −*z*. Symmetry codes for phase 2: (i) 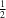 − *x*, −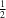 + *y*, 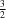 − *z*; (ii) 1 − *x*, −*y*, 1 − *z*; (iii) 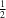 − *x*, 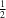 + *y*, 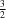 − *z*; (iv) *x*, −1 + *y*, *z*; (v) 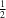 − *x*, −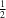 + *y*, 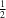 − *z*; (vi) −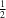 + *x*, 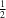 − *y*, 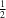 + *z*; (vii) −*x*, 1 − *y*, 1 − *z*.

**Table 2:** Experimental details

	Phase 1	Phase 2
Crystal data		
Chemical formula	C_4_H_8_O_4_S_2_	C_4_H_8_O_4_S_2_
*M*_r_	184.22	184.22
Crystal system, space group	Monoclinic, *C*2/*m*	Monoclinic, *P*2_1_/*n*
Temperature (K)	233	150
*a*, *b*, *c* (Å)	9.073 (8), 7.077 (6), 5.597 (5)	7.1308 (5), 5.7245 (4), 8.3760 (6)
β (°)	105.894 (10)	91.138 (2)
*V* (Å^3^)	345.6 (5)	341.84 (4)
*Z*	2	2
Radiation type	Mo *K*α	Synchrotron, λ = 0.7288 Å
μ (mm^−1^)	0.72	0.78
Crystal size (mm)	0.43 × 0.35 × 0.35	0.04 × 0.03 × 0.02

Data collection		
Diffractometer	APEXII CCD	Bruker D8 Photon-2
Absorption correction	Multi-scan (*SADABS*; Krause *et al.*, 2015 ▸)	Multi-scan (*SADABS*; Krause *et al.*, 2015 ▸)
*T*_min_, *T*_max_	0.747, 0.787	0.811, 0.862
No. of measured, independent and observed [*I* > 2σ(*I*)] reflections	433, 433, 428	14904, 1041, 957
*R*_int_	–	0.036
(sin θ/λ)_max_ (Å^−1^)	0.652	0.714

Refinement		
*R*[*F* ^2^ > 2σ(*F* ^2^)], *wR*(*F* ^2^), *S*	0.023, 0.067, 1.19	0.028, 0.065, 1.11
No. of reflections	433	1041
No. of parameters	37	62
H-atom treatment	All H-atom parameters refined	All H-atom parameters refined
Δρ_max_, Δρ_min_ (e Å^−3^)	0.32, −0.32	0.39, −0.44

Computer programs: *APEX3* and *SAINT* (Bruker, 2016 ▸), *SHELXL* (Sheldrick, 2015 ▸), *OLEX2* (Dolomanov *et al.*, 2009 ▸), *CrystalMaker* (Palmer, 2014 ▸) and *publCIF* (Westrip, 2010 ▸).

## supplementary crystallographic information

### 1,4-Dithiane 1,1,4,4-tetraoxide (14-disulphone-phase1) Crystal data

**Table:**

C_4_H_8_O_4_S_2_	*F*(000) = 192
*M**_r_* = 184.22	*D*_x_ = 1.770 Mg m^−^^3^
Monoclinic, *C*2/*m*	Mo *K*α radiation, λ = 0.71073 Å
*a* = 9.073 (8) Å	Cell parameters from 999 reflections
*b* = 7.077 (6) Å	θ = 3.7–27.7°
*c* = 5.597 (5) Å	µ = 0.72 mm^−^^1^
β = 105.894 (10)°	*T* = 233 K
*V* = 345.6 (5) Å^3^	Irregular block, colorless
*Z* = 2	0.43 × 0.35 × 0.35 mm

### 1,4-Dithiane 1,1,4,4-tetraoxide (14-disulphone-phase1) Data collection

**Table:**

APEXII CCD diffractometer	428 reflections with *I* > 2σ(*I*)
θ/2θ scans	θ_max_ = 27.6°, θ_min_ = 3.7°
Absorption correction: multi-scan (SADABS; Krause *et al.*, 2015)	*h* = −11→11
*T*_min_ = 0.747, *T*_max_ = 0.787	*k* = 0→9
433 measured reflections	*l* = 0→7
433 independent reflections	

### 1,4-Dithiane 1,1,4,4-tetraoxide (14-disulphone-phase1) Refinement

**Table:**

Refinement on *F*^2^	Primary atom site location: dual
Least-squares matrix: full	Secondary atom site location: difference Fourier map
*R*[*F*^2^ > 2σ(*F*^2^)] = 0.023	Hydrogen site location: difference Fourier map
*wR*(*F*^2^) = 0.067	All H-atom parameters refined
*S* = 1.19	*w* = 1/[σ^2^(*F*_o_^2^) + (0.0297*P*)^2^ + 0.2882*P*] where *P* = (*F*_o_^2^ + 2*F*_c_^2^)/3
433 reflections	(Δ/σ)_max_ = 0.001
37 parameters	Δρ_max_ = 0.32 e Å^−^^3^
0 restraints	Δρ_min_ = −0.32 e Å^−^^3^

### 1,4-Dithiane 1,1,4,4-tetraoxide (14-disulphone-phase1) Special details

**Table:**

Geometry. All esds (except the esd in the dihedral angle between two l.s. planes) are estimated using the full covariance matrix. The cell esds are taken into account individually in the estimation of esds in distances, angles and torsion angles; correlations between esds in cell parameters are only used when they are defined by crystal symmetry. An approximate (isotropic) treatment of cell esds is used for estimating esds involving l.s. planes.
Refinement. Refined as a 2-component twin.

### 1,4-Dithiane 1,1,4,4-tetraoxide (14-disulphone-phase1) Fractional atomic coordinates and isotropic or equivalent isotropic displacement parameters (Å^2^)

**Table:**

	*x*	*y*	*z*	*U*_iso_*/*U*_eq_	
S1	0.65320 (6)	0.500000	0.24835 (9)	0.01721 (19)	
O1	0.6955 (2)	0.500000	0.5165 (3)	0.0273 (4)	
O2	0.77466 (18)	0.500000	0.1266 (3)	0.0258 (4)	
C1	0.53198 (17)	0.3028 (2)	0.1408 (3)	0.0198 (3)	
H1A	0.454 (2)	0.305 (3)	0.225 (4)	0.024 (5)*	
H1B	0.590 (2)	0.200 (3)	0.192 (4)	0.028 (5)*	

### 1,4-Dithiane 1,1,4,4-tetraoxide (14-disulphone-phase1) Atomic displacement parameters (Å^2^)

**Table:**

	*U*^11^	*U*^22^	*U*^33^	*U*^12^	*U*^13^	*U*^23^
S1	0.0144 (3)	0.0179 (3)	0.0173 (3)	0.000	0.0010 (2)	0.000
O1	0.0270 (8)	0.0319 (10)	0.0183 (8)	0.000	−0.0017 (7)	0.000
O2	0.0166 (8)	0.0289 (9)	0.0326 (9)	0.000	0.0080 (7)	0.000
C1	0.0193 (7)	0.0158 (7)	0.0217 (8)	−0.0011 (5)	0.0015 (6)	0.0026 (6)

### 1,4-Dithiane 1,1,4,4-tetraoxide (14-disulphone-phase1) Geometric parameters (Å, º)

**Table:**

S1—O1	1.444 (2)	C1—C1^ii^	1.523 (3)
S1—O2	1.4456 (19)	C1—H1A	0.949 (19)
S1—C1	1.7768 (19)	C1—H1B	0.90 (2)
S1—C1^i^	1.7768 (19)		
			
O1—S1—O2	118.03 (11)	S1—C1—H1A	106.9 (12)
O1—S1—C1^i^	108.42 (7)	S1—C1—H1B	105.9 (13)
O1—S1—C1	108.42 (7)	C1^ii^—C1—S1	112.07 (9)
O2—S1—C1^i^	108.74 (8)	C1^ii^—C1—H1A	113.1 (11)
O2—S1—C1	108.74 (8)	C1^ii^—C1—H1B	110.6 (13)
C1—S1—C1^i^	103.52 (12)	H1A—C1—H1B	108.0 (17)
			
O1—S1—C1—C1^ii^	174.03 (12)	C1^i^—S1—C1—C1^ii^	59.03 (17)
O2—S1—C1—C1^ii^	−56.47 (15)		

Symmetry codes: (i) *x*, −*y*+1, *z*; (ii) −*x*+1, *y*, −*z*.

### 1,4-Dithiane 1,1,4,4-tetraoxide (14-disulphone-phase2) Crystal data

**Table:**

C_4_H_8_O_4_S_2_	*F*(000) = 192
*M**_r_* = 184.22	*D*_x_ = 1.790 Mg m^−^^3^
Monoclinic, *P*2_1_/*n*	Synchrotron radiation, λ = 0.7288 Å
*a* = 7.1308 (5) Å	Cell parameters from 4318 reflections
*b* = 5.7245 (4) Å	θ = 29.1–3.8°
*c* = 8.3760 (6) Å	µ = 0.78 mm^−^^1^
β = 91.138 (2)°	*T* = 150 K
*V* = 341.84 (4) Å^3^	Tablet, colorless
*Z* = 2	0.04 × 0.03 × 0.02 mm

### 1,4-Dithiane 1,1,4,4-tetraoxide (14-disulphone-phase2) Data collection

**Table:**

Bruker D8 Photon-2 diffractometer	1041 independent reflections
Radiation source: synchrotron	957 reflections with *I* > 2σ(*I*)
Detector resolution: 10.34 pixels mm^-1^	*R*_int_ = 0.036
φ and ω scans	θ_max_ = 31.4°, θ_min_ = 3.8°
Absorption correction: multi-scan (SADABS; Krause *et al.*, 2015)	*h* = −10→10
*T*_min_ = 0.811, *T*_max_ = 0.862	*k* = −8→8
14904 measured reflections	*l* = −11→11

### 1,4-Dithiane 1,1,4,4-tetraoxide (14-disulphone-phase2) Refinement

**Table:**

Refinement on *F*^2^	Primary atom site location: dual
Least-squares matrix: full	Secondary atom site location: difference Fourier map
*R*[*F*^2^ > 2σ(*F*^2^)] = 0.028	Hydrogen site location: difference Fourier map
*wR*(*F*^2^) = 0.065	All H-atom parameters refined
*S* = 1.11	*w* = 1/[σ^2^(*F*_o_^2^) + (0.0197*P*)^2^ + 0.3362*P*] where *P* = (*F*_o_^2^ + 2*F*_c_^2^)/3
1041 reflections	(Δ/σ)_max_ = 0.001
62 parameters	Δρ_max_ = 0.39 e Å^−^^3^
0 restraints	Δρ_min_ = −0.44 e Å^−^^3^

### 1,4-Dithiane 1,1,4,4-tetraoxide (14-disulphone-phase2) Special details

**Table:**

Geometry. All esds (except the esd in the dihedral angle between two l.s. planes) are estimated using the full covariance matrix. The cell esds are taken into account individually in the estimation of esds in distances, angles and torsion angles; correlations between esds in cell parameters are only used when they are defined by crystal symmetry. An approximate (isotropic) treatment of cell esds is used for estimating esds involving l.s. planes.

### 1,4-Dithiane 1,1,4,4-tetraoxide (14-disulphone-phase2) Fractional atomic coordinates and isotropic or equivalent isotropic displacement parameters (Å^2^)

**Table:**

	*x*	*y*	*z*	*U*_iso_*/*U*_eq_	
S1	0.19891 (5)	0.14315 (6)	0.54564 (4)	0.01294 (10)	
O1	0.35411 (15)	0.14742 (19)	0.65934 (13)	0.0194 (2)	
O2	0.19584 (15)	0.31562 (19)	0.41982 (12)	0.0186 (2)	
C1	0.1857 (2)	−0.1390 (2)	0.45787 (17)	0.0149 (3)	
C2	−0.0137 (2)	0.1639 (3)	0.65211 (17)	0.0147 (3)	
H1A	0.186 (3)	−0.247 (4)	0.541 (2)	0.024 (5)*	
H1B	0.299 (3)	−0.156 (3)	0.401 (2)	0.020 (5)*	
H2A	−0.006 (3)	0.049 (3)	0.734 (2)	0.016 (4)*	
H2B	−0.008 (3)	0.314 (4)	0.696 (2)	0.024 (5)*	

### 1,4-Dithiane 1,1,4,4-tetraoxide (14-disulphone-phase2) Atomic displacement parameters (Å^2^)

**Table:**

	*U*^11^	*U*^22^	*U*^33^	*U*^12^	*U*^13^	*U*^23^
S1	0.01212 (16)	0.01260 (16)	0.01412 (17)	−0.00078 (11)	0.00029 (11)	−0.00062 (12)
O1	0.0153 (5)	0.0218 (5)	0.0208 (5)	−0.0010 (4)	−0.0042 (4)	−0.0031 (4)
O2	0.0209 (5)	0.0156 (5)	0.0194 (5)	−0.0018 (4)	0.0024 (4)	0.0038 (4)
C1	0.0135 (6)	0.0134 (6)	0.0177 (6)	0.0010 (5)	0.0011 (5)	−0.0018 (5)
C2	0.0146 (6)	0.0157 (6)	0.0137 (6)	−0.0002 (5)	0.0013 (5)	−0.0022 (5)

### 1,4-Dithiane 1,1,4,4-tetraoxide (14-disulphone-phase2) Geometric parameters (Å, º)

**Table:**

S1—O1	1.4459 (11)	C1—H1A	0.93 (2)
S1—O2	1.4439 (11)	C1—H1B	0.95 (2)
S1—C1	1.7762 (14)	C2—H2A	0.952 (19)
S1—C2	1.7778 (14)	C2—H2B	0.94 (2)
C1—C2^i^	1.5265 (19)		
			
O1—S1—C1	108.75 (7)	C2^i^—C1—H1A	112.1 (12)
O1—S1—C2	108.51 (7)	C2^i^—C1—H1B	111.5 (11)
O2—S1—O1	118.00 (7)	H1A—C1—H1B	108.6 (16)
O2—S1—C1	108.65 (7)	S1—C2—H2A	106.2 (11)
O2—S1—C2	108.65 (7)	S1—C2—H2B	103.3 (12)
C1—S1—C2	103.29 (7)	C1^i^—C2—S1	111.98 (10)
S1—C1—H1A	107.1 (13)	C1^i^—C2—H2A	113.8 (11)
S1—C1—H1B	105.4 (12)	C1^i^—C2—H2B	110.6 (12)
C2^i^—C1—S1	111.72 (10)	H2A—C2—H2B	110.4 (16)

Symmetry code: (i) −*x*, −*y*, −*z*+1.
